# Summation of activation at the branch-stem transition of *Mimosa pudica*; a comparison with summation in cardiac tissue

**DOI:** 10.1371/journal.pone.0286103

**Published:** 2023-05-19

**Authors:** Jacques M. T. de Bakker, Ruben Coronel

**Affiliations:** Department of Experimental Cardiology, Heart Center, Academic Medical Center, Amsterdam, The Netherlands; National Institute of Environmental Health Sciences National Toxicology Program Division, UNITED STATES

## Abstract

In *Mimosa pudica* plants, local and global responses to environmental stimuli are associated with different types of electrical activity. Non-damaging stimuli (e.g. cooling) generate action potentials (APs), whereas damaging stimuli (e.g. heating) are associated with variation potentials (VPs). Local cooling of *Mimosa* branches resulted in APs that propagated up to the branch-stem interface and caused drooping of the branch (local response). This electrical activation did not pass the interface. If the branch was triggered by heat, however, a VP was transferred to the stem and caused activation of the entire plant (global response). VPs caused by heat were always preceded by APs and summation of the two types of activation appeared to be necessary for the activation to pass the branch-stem interface. Mechanical cutting of leaves also resulted in VPs preceded by APs, but in those cases a time delay was present between the two activations, which prevented adequate summation and transmission of activation. Simultaneous cold-induced activation of a branch and the stem below the interface occasionally resulted in summation sufficient to activate the stem beyond the interface. To investigate the effect of activation delay on summation, a similar structure of excitable converging pathways, consisting of a star-shaped pattern of neonatal rat heart cells, was used. In this model, summation of activation was not hindered by a small degree of asynchrony. The observations indicate that summation occurs in excitable branching structures and suggest that summation of activation plays a role in the propagation of nocuous stimuli in *Mimosa*.

## Introduction

*Mimosa pudica* plants react differently to non-damaging (e.g. cooling, touching) or damaging (e.g. heating, cutting) stimuli. The response involves closing of pinnules (leaves) and/or bending of petioles (branches) at the pulvinus (the swelling at the base of a petiole). The extent of the response depends on whether the stimulus is damaging or non-damaging. Heating leaves or petioles usually results in a drooping of all leaves and petioles of the plant, whereas the reaction is limited exclusively to the stimulated petiole after cooling or light touch. To accomplish these feats, *Mimosa* uses electrophysiological mechanisms for information perception, transmission and processing. These mechanisms include the genesis of propagating action potentials (APs), variation potentials (VPs) and system potentials (SPs) [1–3]. APs have a duration of several tens of seconds and are related to non-damaging factors. VPs have durations of minutes and are caused by damaging factors. SPs are transient hyperpolarizations with a duration of tens of minutes. induced by chemical cascade responses at the site of damage. APs are primarily based on Ca^2+^, Cl^-^, and K^+^ transmembrane currents, although transient H^+^-ATPase inactivation may also play a role [4, 5]. The origin of VPs is less clear, but traditionally connected with H^+^-ATPase inactivation. Several studies using inhibitors of H^+^-ATPase and pH changes show that changes in H^+^-ATPase activity can affect VP generation [2, 6–12]. Other studies show that Ca^2+^ influx [7, 9, 12]and Cl^-^ channel activation may also be involved in VP events [13]. Lastly, SPs are likely connected to H^+^-ATPase activation [14].

Bending of petioles and folding of pinnules are the direct visible reactions of *Mimosa* to external stimuli that are initiated by propagation of electrical activity. Plants are, however, affected by a great variety of abiotic and biotic stimuli, that are communicated by electrical signals. These signals affect different physiological processes including gene expression [15–17] and photosynthesis [18–21], but there are many others that are extensively described and discussed by Sukhova and Sukhov [22, 23]. Interestingly, these authors also hypothesize that even programmed cell death in plant cells is likely connected with electrical signals, and they provide various arguments to support this hypothesis.

Stimulation of a petiole with a drop of ice-cold water (~ 4°C) resulted in propagation of the electrical impulse (AP) along the petiole, but activation was always blocked at the intersection toward the stem (at the primary pulvinus). In contrast, short heating of pinnules or petioles resulted in activation that overtook the pulvinus-stem passage. The related VP activation was, however, always preceded by AP activation. Because major pathways for propagation of APs in *Mimosa* are the phloem vessels, whereas VPs have been shown to arise in parenchymal cells near xylem vessels, APs and VPs may follow different pathways, which could lead to summation of activation.

In excitable cardiac tissue, a sudden increase in the number of excitable cells along the course of the activation path (at a tissue discontinuity) has been shown to result in conduction delay or activation block (complete or partially) in specific geometrical structures. This may occur at sites where a myocardial bundle suddenly widens or two or more bundles merge [24, 25]. In the second case, single bundle activation may be blocked at the connection, because at that site, current must be delivered to multiple other bundles. Cardiac activation block can be overcome in three ways: 1) prolongation of the AP duration may cause the tissue distal to the bifurcation to reach activation threshold, 2) if two bundles are activated concurrently and activation arrives nearly simultaneously at the connection, activation may pass by means of summation, 3) reduced cell-to-cell coupling at the downstream site of the tissue discontinuity may give rise to paradoxical improvement of impulse conduction [26–28].

Because continuation of electrical propagation at the transition from petiole to stem is associated with two activations (AP and VP), which may follow different pathways, we hypothesized that summation of AP and VP may be necessary and sufficient for the electrical signal to pass the transition between petiole and stem in *Mimosa*. Our data show that summation of activation can occur and promote transference of activation at the intersection between petiole and stem. The observations further suggest that a small degree of asynchrony between AP and VP activation does not prevent summation. To substantiate the effect of asynchrony between two activations on summation we made branching structures of neonatal rat cardiomyocytes to investigate summation of activation at different degrees of asynchrony.

## Results

### Electrogram characteristics of cold- and heat-induced activation

**Fig 1:**
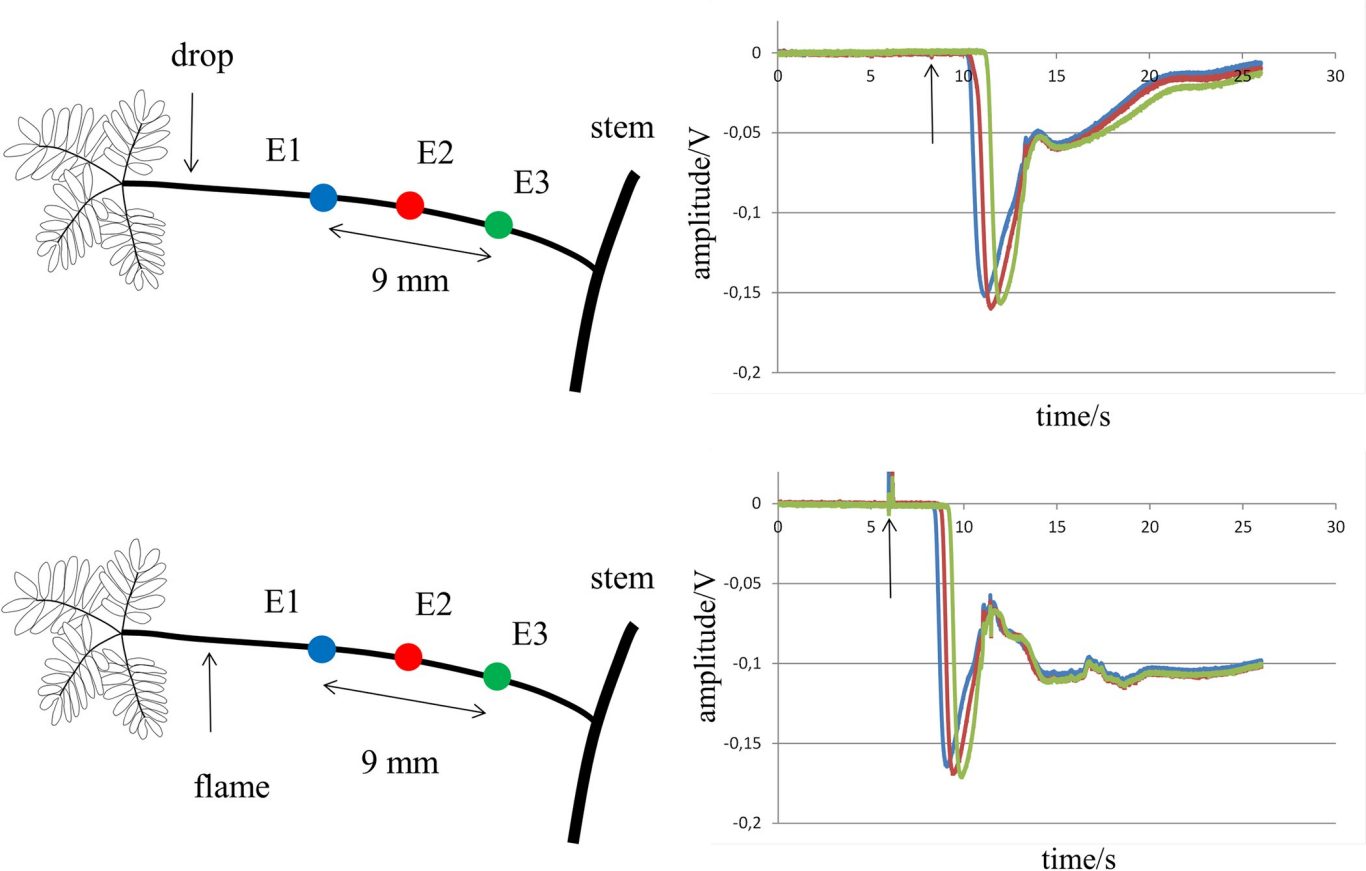
Electrogram recordings at a petiole after cold- and heat-induced activation of the petiole. Upper panel: tracings are electrograms recorded from a petiole after activation with drops of cold water at the site indicated at the left. Colors of the electrograms correspond with the colors of the recording sites at the petiole. Lower panel: tracings are electrograms recorded at the same sites as in the upper panel after activation of the petiole by a flame, as indicated in the left panel. Arrows in the right panels indicate time of action of stressors.

Unipolar extracellular electrograms were predominantly negative, independent of the mode of stimulation. In sporadic cases a tiny initial positive deflection was observed. Activation of the petiole with a drop of cold water resulted in electrograms with relatively short duration (several tens of seconds) and amplitudes around -0.1 V. Flame-induced activation from the same site of the petiole or leaves resulted in electrograms with a similar initial morphology, but with a much longer duration (minutes) (Fig 1). To determine differences in unipolar electrograms for cold- or heat-induced activation, recordings were made from the same 3 sites in 5 plants during both interventions (n = 15). In 7 measurements, recording sites were arranged in a row on the petioles as in Fig 1, whereas one of the electrode terminals was on the primary pulvinus in 8 cases.

**Fig 2:**
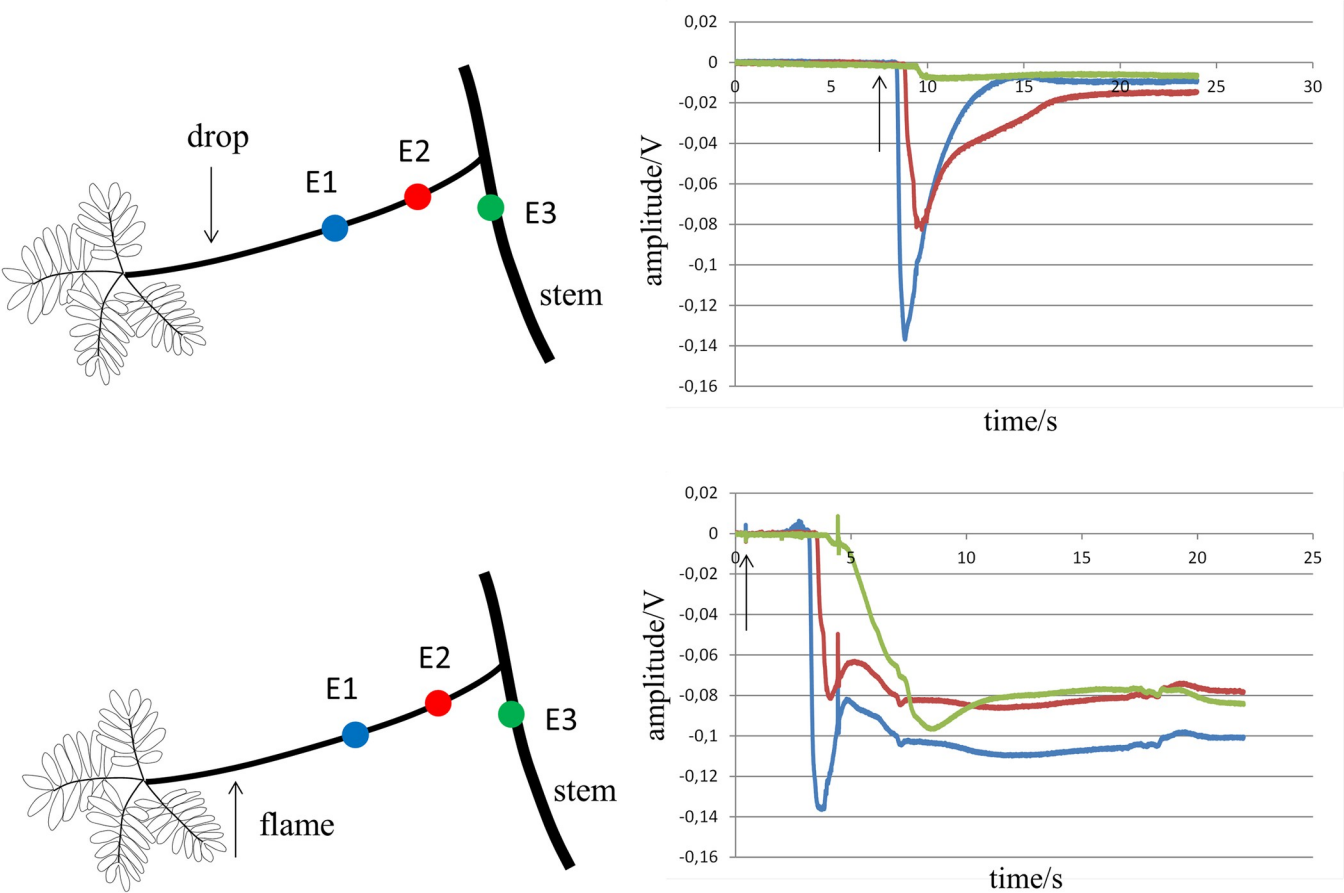
Electrogram recordings at a petiole and stem after cold- and heat-induced activation of the petiole. Upper panel: tracings are unipolar extracellular recordings made at 2 sites of a petiole (E1 and E2) and 1 site at the stem (E3), as indicated in the left panel. The petiole was activated with a drop of cold water (downward arrow). Activation is blocked at the petiole-stem interface. E3 at the stem only reveals a tiny remote deflection (-0.005 V). In the lower panel the petiole is activated by a flame (upward arrow). The initial deflections of the electrograms at E1 and E2 are similar to those observed during cold-induced activation. Depolarizations, however, last much longer. In addition, an electrogram is recorded at E3 (-0.1 V), indicating that the stem is activated (green tracing). Arrows in the right panels indicate time of action of stressors.

Eight measurements were made with 2 electrode terminals attached to the petiole and 1 affixed to the stem, 8 mm above or below the branch attachment (Fig 2). Electrode E3 was positioned at the stem distal from the pulvinus. As in Fig 1, the initial phase of electrograms E1 and E2 recorded from the petiole was similar for cold- (upper panel) and heat- (lower panel) induced activation. During cold-induced activation, the duration of the electrogram was short (a few seconds) and activation block occurred at the interface between petiole and stem. The electrogram at E3 only showed a deflection with low amplitude, representing remote activity detection. During heat-induced activation, the initial phase of the electrograms E1 and E2 looked similar to those observed during cold-induced activation, but they were followed by a sustained secondary component. The resulting activity was now of greater duration (lower panel). Activation passed the intersection as evidenced by the prominent electrogram present at E3. This electrogram also had a long duration (minutes), as with the signals at E1 and E2.

Table 1 shows the maximum negative amplitude, dV/dt minimal and conduction velocity at the petiole for cold- and heat-induced activation. These values always occurred during the first phase of the electrograms. Data show that these parameters were not statistically different for cold- and heat-induced activation.

**Table 1:** Electrogram parameters and conduction velocity after cold- and heat-induced activation.

	Cold induced activation	Heat induced activation	p-value
Amplitude/min E1	- 0.126 ±0.026 V	- 0.118 ±0.023 V	0.29
Amplitude/min E2	- 0.099 ±0.043 V	- 0.097 ±0.034 V	0.70
dV/dt min E1	- 0.498 ±0.30 V/s	- 0.482 ±0.32 V/s	0.69
dV/dt min E2	- 0.343 ±0.26 V/s	- 0.343 ±0.26 V/s	0.99
CV E1 –E2	1.9 ±1.3 cm/s	2.0 ±1.0 cm/s	0.48

Recordings were made at E1 and E2 (Fig 1) from 15 petioles of 5 *Mimosa* plants. Amplitude/min is the maximal negative amplitude of electrograms. dV/dt min is the maximal negative value of the derivative of electrograms. CV is the conduction velocity of activation between E1 and E2. ± values are standard deviations. P-values are paired T-tests for differences between cold- and heat-induced values and indicate that there are no significant differences between the electrogram parameters and conduction velocity for cold- and heat-induced activation.

Whereas parameters of the initial deflections of the electrograms were similar for cold- and heat-induced electrograms and fit with the characteristics of APs, the long duration of the electrograms after heating is more compatible with VP activation. Data obtained during cold-induced activation suggested that VP activation was preceded by AP activation. During flame-induced activation, the initial AP-like potential was always followed by a VP within a short time (a few seconds). We did not observe a large time delay between the two types of activation in our measurements after heating.

### Electrogram characteristics after mechanically cutting a leaf

**Fig 3:**
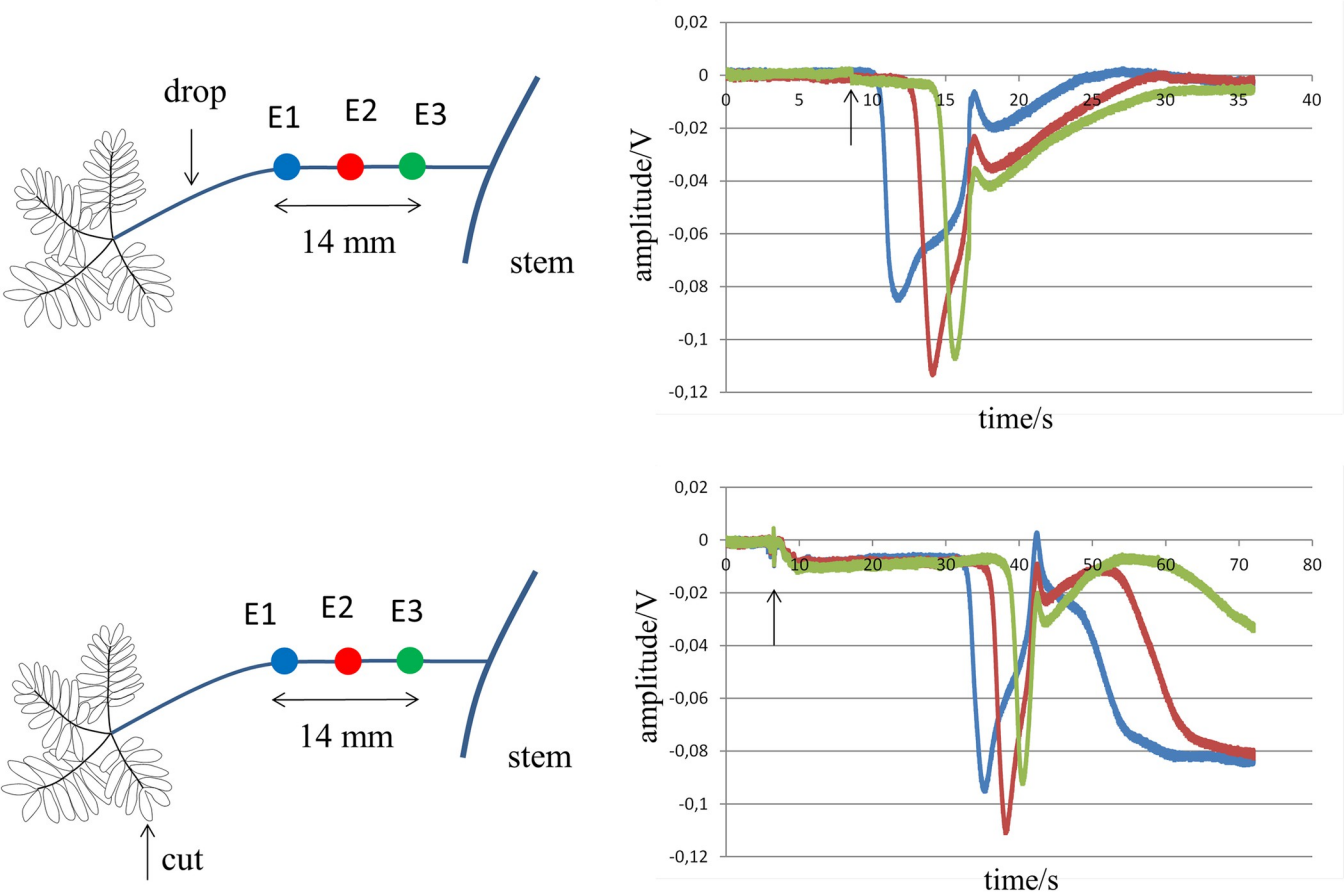
Electrograms recorded at a petiole after activation induced by a cold drop applied to the petiole and cutting a leaf. Upper panel: tracings are unipolar extracellular electrograms recorded from a petiole after activating the petiole with a drop of cold water. Activation induced by the water drop resulted in short lasting electrograms (about 10 seconds). Lower panel: tracings are electrograms generated after mechanically cutting leaves with a scissor. The electrograms are initially similar to those after cold-induced activation. However, they are followed by a second depolarization with long duration after a delay of approximately 20 seconds. Arrows in the right panels indicate time of action of stressors.

Additional recordings were made after mechanically cutting a leaf—another damaging factor. This intervention, however, frequently showed a time delay of several tens of seconds between the two components (AP and VP) of the electrograms. An example is shown in Fig 3.

The upper panel shows electrograms at 3 sites on the petiole after cold drop-induced activation. In the lower panel. a leaf of the same petiole was mechanically cut. The initial deflection of the electrograms after cutting is similar to the AP-related electrograms in the upper panel. After several tens of seconds, the initial deflection is followed by a VP component. Only the cut petiole drooped—activation did not proceed to the stem and other petioles. In 5 experiments in which activation was provoked by cutting leaves, a delay between AP and VP of 10 to 25 seconds was observed and activation failed to continue to other parts of the plant. This contrasts heat- induced activation, where no large time delay between AP and VP was seen and activation was able to pass the branch-stem interface. The observations suggest that summation of AP and VP activation at an appropriately short delay (and of sufficient amplitude) is necessary to allow passage of the activation wave across the intersection between petiole and stem.

### Summation of activation in *Mimosa*

**Fig 4:**
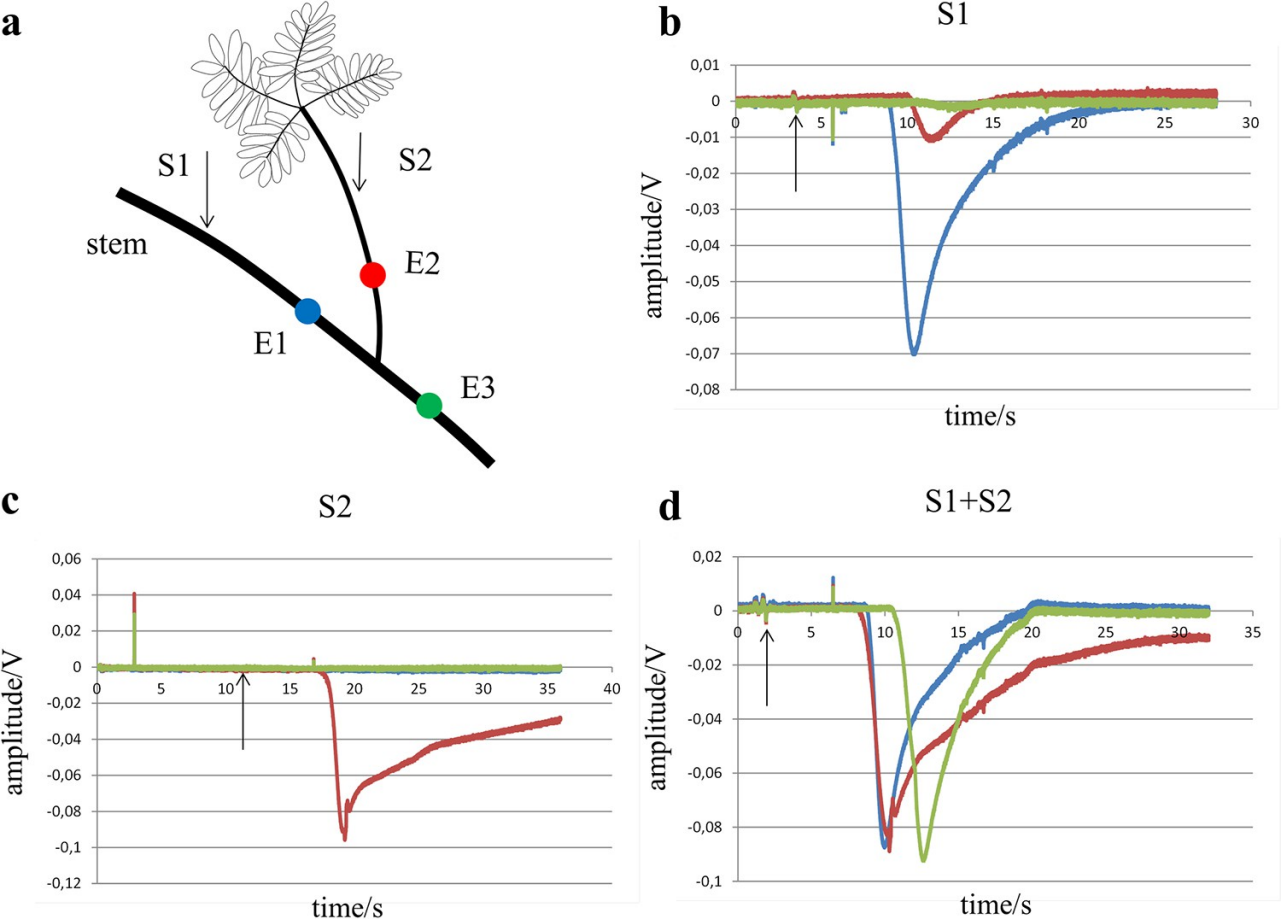
Summation of activation after stimulating stem and petiole with a drop of cold water. Panel **a** shows the distribution of the electrode (E1-E3) and stimulation (S1, S2) sites. Stimulation was achieved by a drop of cold water. Panel **b** shows the signals at the electrodes after activating the stem at site S1. Only at E1, an electrogram compatible with local activation is present. In panel **c** the petiole is activated at S2. Only tracing E2 reveals local activation. Panel **d** shows electrograms after stimulation at S1 and S2 simultaneously. Local activation is present at all three recording sites, indicating propagation towards E3 on the stem. Arrows in the right panels indicate time of action of stressors.

We next tested whether the intersection between petiole and stem represents a site of tissue discontinuity where activation from one limb blocks, but activation from two limbs at an appropriate delay can allow for transfer of activation. This was tested in 19 experiments. Activation was initiated by drops of cold water at the petiole and/or stem. Electrodes were attached at the following positions: 1) at the stem 8 mm before a branching point, 2) at the petiole that sprouted from the branching point and 3) at the stem 8 mm beyond the branching point (see Fig 4). Before assessing summation of activation, it was tested whether activation from position S1 at the stem was blocked at the intersection. Although stimulation of a petiole by drops of cold water in the previous recordings always blocked toward the stem, this was also tested here (stimulation at S2).

Panel **d** of Fig 4 shows summation of activation after the simultaneous activation of a site at the stem (S1) and a site at the petiole (S2). The arrangement of the electrodes is depicted in panel **a**. Activation of site S1 of the stem alone gave rise to a deflection (amplitude -0.07 V) at site E1 distal from S1 (panel **b**). Activation toward E2 on the petiole and E3 on the stem was blocked. No electrogram was recorded at E3 (flat green line) and the small signal at E2 (-0.01 V) is electrotonic (the petiole did not bend). Activation of the petiole at S2 only gave rise to an electrogram at E2, which was expected from previous recordings (panel **c**). The simultaneous activation of S1 and S2 resulted in electrograms at all 3 recording sites. This indicated that summation of activation arriving at the petiole-branch transition was able to pass the bifurcation and activate the distal part of the stem (E3). Of the 19 experiments carried out to test whether summation was possible, summation was observed in 5. In 3 experiments summation could not be tested because stimulation of E1 resulted already in activation of site E3. In the other cases of failed summation, it is likely that the time difference between activation of the stem and petiole was too long.

Mean time delay for activation at the petiole and stem for conduction and conduction block was 753±669 ms and 1956±559 ms respectively for paired data (recordings performed in the same plant). Time delay was roughly a factor 2.5 shorter for cases where conduction occurred. The difference is statistically significant (p = 0.017).

Summation of activation in our study occurred during both heating and cooling. Heating resulted in an AP together with a VP. If they occurred simultaneously, or with a small delay, summation occurred resulting in activation that was able to pass the petiole-stem interface. Cooling only generated an AP and could not pass the interface. However, if cooling was applied to the petiole and the proximal part of the stem, the two generated APs could sum together and activate the distal part of the stem. Heat can occur under natural conditions, cooling by a drop of ice water is an artificial stressor, but the results show that activations of the same type (APs) can also summate in plants. We speculate that temporal and/or spatial summation can be beneficial to the plant, to provide a commensurate (local, generalized) response to a series of environmental stressors.

### Summation in cardiac ventricular tissue

**Fig 5:**
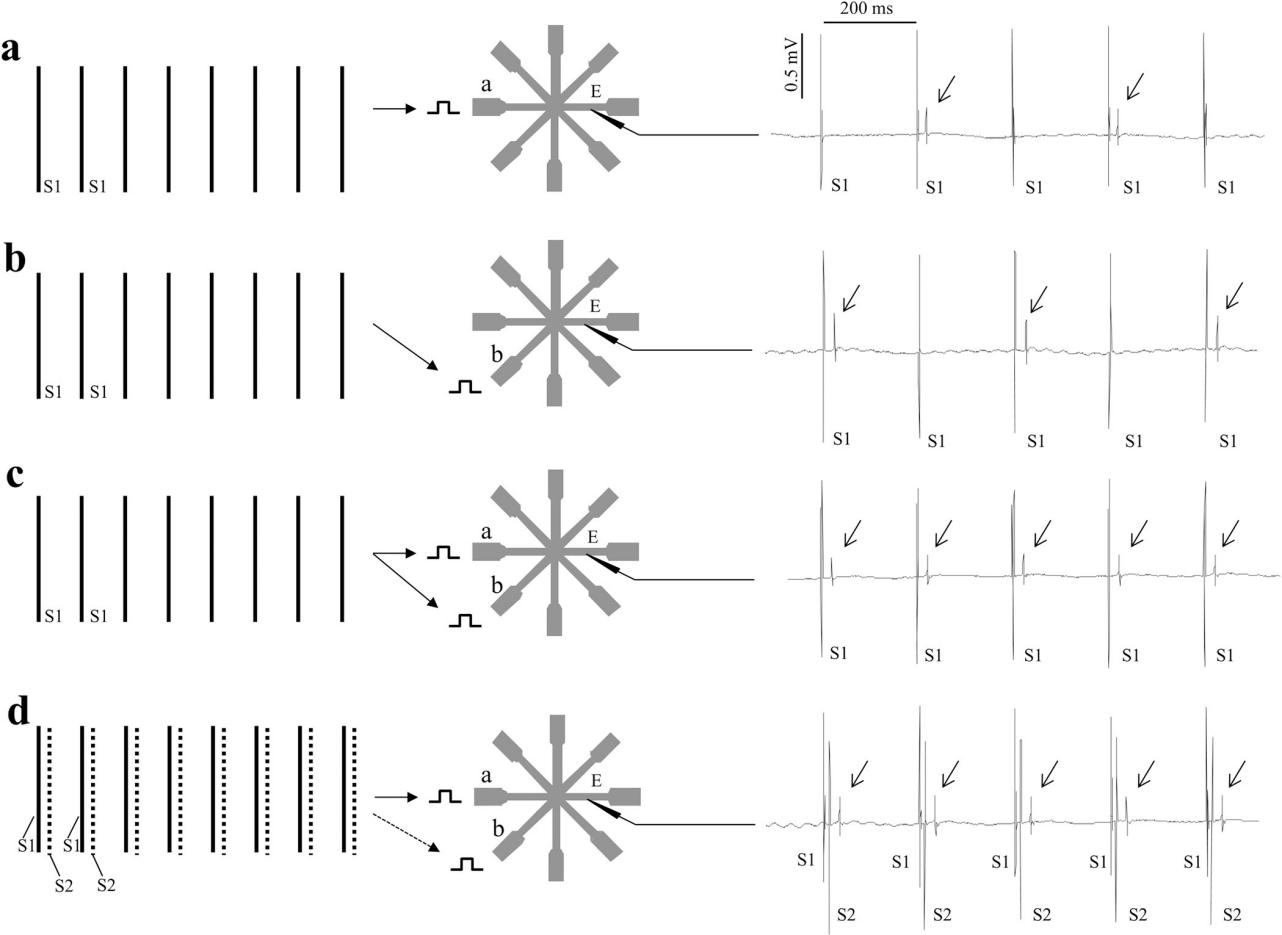
Summation of activation in a star-shaped structure of cardiomyocytes. The left panel shows the sequence of stimuli. S1 are basic stimuli, S2 are early coupled stimuli. The middle panel shows the star-shaped pattern of cardiomyocytes. The right panels show unipolar electrograms at site E for different stimulation modes. Vertical lines marked S1 and S2 in the right panels are stimulus artifacts and arrows in the tracings point to activation at recording site E. In panel **a** tract a is stimulated (marker) with a cycle length that resulted in 2:1 conduction block. In panel **b** a neighboring tract b is stimulated (marker) at the same cycle length, that also resulted in 2:1 conduction block. In panel **c** tracts a and b are stimulated simultaneously with the same cycle length, which resulted in 1:1 conduction. In panel **d** tracts a and b are stimulated with the same cycle length as before, but S1 and S2 had a time delay of 30 ms. This stimulation protocol also resulted in 1:1 conduction. For larger delays, 2:1 block occurred.

For summation of activation in cardiac tissue, star-shaped patterns of coupled neonatal rat cardiomyocytes were used, as shown in Fig 5 (middle panel). Summation of activation was tested by stimulating two neighboring tracts at a cycle length that resulted in 2:1 conduction block for both tracts (n = 6). Pacing of the two tracts was performed: 1) simultaneously and 2) by using a time delay between the stimuli. Panels **a** and **b** of Fig 5 show that stimulation of tract a and b separately at that cycle length resulted in 2:1 block. At electrode E, an electrogram is present every second stimulus. In panel **c** the two tracts are stimulated simultaneously, which resulted in 1:1 activation at site E. Both tracts are also stimulated in panel **d**, but here a time delay of 30 ms was used. There is still summation (1:1 conduction), but for delays > 30 ms, 2:1 activation block occurred.

## Discussion

In the current study we recorded unipolar extracellular electrograms from petioles, pulvini and the stem of *Mimosa pudica* plants to determine spread of activation induced by different stimuli, whether noxious or innocuous. We showed that: 1) unipolar extracellular electrograms from *Mimosa* petioles and stems are mainly negative only, 2) characteristics of the initial phase of the electrograms is independent of the mode of activation (cold or heat) of the petiole, suggesting that APs precede VPs during heat-induced activation, 3) activations induced by cold at a petiole and stem are able to sum to proceed activation beyond the stem-petiole intersection, 4) activation of APs and VPs may summate to support the passage of activation at the intersection between petiole and stem, 5) summation of activation in converging structures of cardiac cells revealed that a small degree of asynchrony between two wave fronts at the converging point is not detrimental to summation.

Stimulation of a petiole with a drop of ice-cold water (~ 4°C) resulted in propagation of the electrical impulse along the petiole, but activation was always blocked at the intersection toward the stem (at the primary pulvinus). In contrast, short heating of pinnules or petioles resulted in activation that crossed the petiole-stem passage and led to a more general response. Cold-induced activation has been shown to result in APs based on the all-or-none law as in nerve cells and muscle fibers with relatively short duration of the AP [29, 30]. Heating-induced VPs show a long duration of the AP, and in contrast to APs the amplitude of VPs reduces with increasing distance from the site of generation [9, 10, 31]. Houwink showed that the initial morphology of the electrograms suggests that VPs are preceded by APs [29]. These observations made by other investigators are compatible with those obtained in our study.

The question arises whether VP activation alone is sufficient to pass the petiole-stem interface or that summation of AP and VP activation, prolonged AP duration (APD), or reduced coupling is needed. The geometry of the petiole-stem interface is in favor of a summation effect. The sole increase in APD during VP is unlikely to cause the transition of activation at the interface because mechanically cutting leaves also causes VPs that are preceded by APs. However, there is a large delay between the two electrical phenomena and activation does not pass the interface. A sudden change in cell-to-cell coupling is also unlikely because of the fast transition of activation from petiole to stem (seconds). Forisomes and callose that are generated by wound response need tens of seconds and many minutes, respectively, to accomplish occlusion of sieve plate pores and/or desmoplasmata in *Vicia faba* [32]. In addition, a reduced coupling must be local; it should only be present at the distal side of the discontinuity.

Therefore, we argue that summation is the most likely explanation. Several studies demonstrate that the major pathway for propagation of APs in *Mimosa* are the phloem vessels [1, 33, 34]. VPs have been shown to be generated at the plasma membrane of parenchymal cells adjacent to xylem vessels. However, Lautner et al. also recorded VPs in sieve elements [35]. At the interface, where the vascular bundles of the petiole meet the vascular bundles of the stem, current to load mismatch may occur. This phenomenon arises in geometrical structures where more current for activation is needed than can be delivered by an upstream activating wave front. The petiole-stem interface represents such a geometry, because the number of cells that must be activated suddenly increases at that site, which requires more current. In addition, the pulvinus comprises next to a central vascular core two layers of excitable flexor and extensor cells that may require additional current to be activated [36]. Thus, activation arriving from the petiole must deliver extra current to activate the increased number of cells for further transference of the electrical activity toward the stem. This additional current can be obtained by the summation of AP and VP.

### Morphology of the unipolar electrogram

Extracellular unipolar electrograms of *Mimosa* plants were already recorded by Houwink in 1935 using tube amplifiers and hook-shaped electrodes attached to petioles [29]. The author also observed that the main deflection of the unipolar electrogram was mainly negative. A tiny initial positive deflection was found in some of the recordings as well as a prominent delay between AP and VP deflections after cutting leaves, compatible with our observations.

The observation that the electrograms in *Mimosa* petioles are virtually negative only at sites where activation fronts pass the electrode differs from the morphology of electrograms in cardiac tissue. In electrograms recorded from healthy myocardial tissue at sites where activation passes the electrode, the morphology is biphasic. The initial deflection is positive and is followed by a negative. The positive and negative deflections have similar amplitudes at sites distant from the site where activation arises. The positive deflection is caused by a capacitive current that occurs during propagation of the AP. This transmembrane current is determined by: I_c_ = C_m_. dV_m_/dt. C_m_ is the membrane capacitance of a cell, which is about 1 μF/cm^2^ for both heart and plant cells [37, 38], dV_m_/dt is the upstroke velocity of the transmembrane potential during an AP. For cardiomyocytes of the working myocardium, this value has been reported to be 297 V/s [39]. In contrast, the upstroke velocity of the AP of *Mimosa pudica* is only 0.2 V/s (derived from [1]). Because the upstroke velocity of *Mimosa* cells is much lower than that of cardiac cells, the capacitive current for *Mimosa* cells is correspondingly small in comparison, which may explain the absence of an initial positive deflection in most cases.

The negative deflections in cardiac and *Mimosa* cells are caused by ionic currents. Whereas the current is carried by sodium ions in heart tissue, calcium and chloride ions are the major contributors to APs in *Mimosa* [40–42]. For VPs the contributors are less clear, but H^+^-ATPase has been reported to play a role [2, 6].

### AP and VP in *Mimosa*

Three types of electric signals have been described in higher plants. The generation of APs involves Ca^2+^ influx and Cl^-^ efflux for depolarization, and K^+^ efflux for the repolarization phase. A second phase of repolarization has been attributed to H^+^-ATPase. Although the H^+^ pump is inactivated by Ca^+^ influx, contribution to the development of AP is small [5]. The second type of electric signal is the VP, which has been associated with a transitional change in the activity of the H^+^-ATPase pump as a major mechanism of depolarization [13, 14, 43, 44]. There is, however, evidence that passive ion flows of Ca^2+^, Cl^-^ and K^+^ also contribute to VP generation [10, 12, 45]. A third mechanism is the SP, which is a transient hyperpolarization related to H^+^-ATPase activation [14]. The role of this type of electric signal is less known.

Propagation of electrical activity is important, because the information obtained by an external interference must be communicated to other sites of the plant to prepare for countermeasures to the distant environmental incident. AP propagation is an active process that fits the cable equation similar to propagation of electrical activity in heart tissue and nerves. The main transmission system in higher plants is formed by conducting bundles [1, 33, 34]. Different tissues are involved, including xylem vessels, sieve elements and parenchyma cells. Intracellular recordings have shown APs in parenchymal cells as well as sieve elements [1]. The two paths have different electrophysiological characteristics in regard to excitability and electrical connection and it is suggested that both paths are used for propagation [3]. Because of the similarity of AP propagation in higher plants and cardiac and nerve tissue, it is likely that structural discontinuities where current supply does not match the amount of current needed to activate cells distal from the discontinuity, will affect transmission of activation.

Propagation of VPs is more complex. Active spreading via local current, chemical wound response cascades, and hydraulic waves have been suggested [14]. Although the chemical and hydraulic hypothesis are both supported by various studies, several problems with both theories remain. Therefore, a combination of the two has been suggested to overcome the weaknesses [46]. Although xylem vessels play a role in VPs, this electrical signal has also been measured in sieve elements [35]. It was suggested that they can pass through the plasmodesmal network and reach the phloem pathway. Because VPs and APs use, to a certain extent, the same pathway, it is not clear how different pathways are used for summation in *Mimosa*. In addition, we cannot exclude that the propagation speed of VPs and APs were different, leading to asynchronous activation at the bifurcation sites.

### Summation of activation in heart tissue

In heart cells, depolarization is carried by sodium ions that generate a fast upstroke of the AP from– 90 mV to + 20 mV within a few milliseconds in normal working myocardium. The AP duration is approximately 200 ms. Activated cells deliver current to their inactivated neighbors to depolarize these cells and thereby provide spread of activation. The amount of current that is needed for propagation depends on the number of cells that have to be activated. In a single strand of myocardial cells this number is rather constant. The number of cells, however, suddenly increases at sites where two myocardial bundles converge. At those merging sites, the depolarizing current delivered by cells of the activated bundle must give enough current to activate the subsequent two bundles. If the current safety factor of a single bundle is too low, not enough current is available to activate the converging site and activation block occurs. Next to the amount of current, cell-to-cell coupling at the interface may also play a role [28].

**Fig 6:**
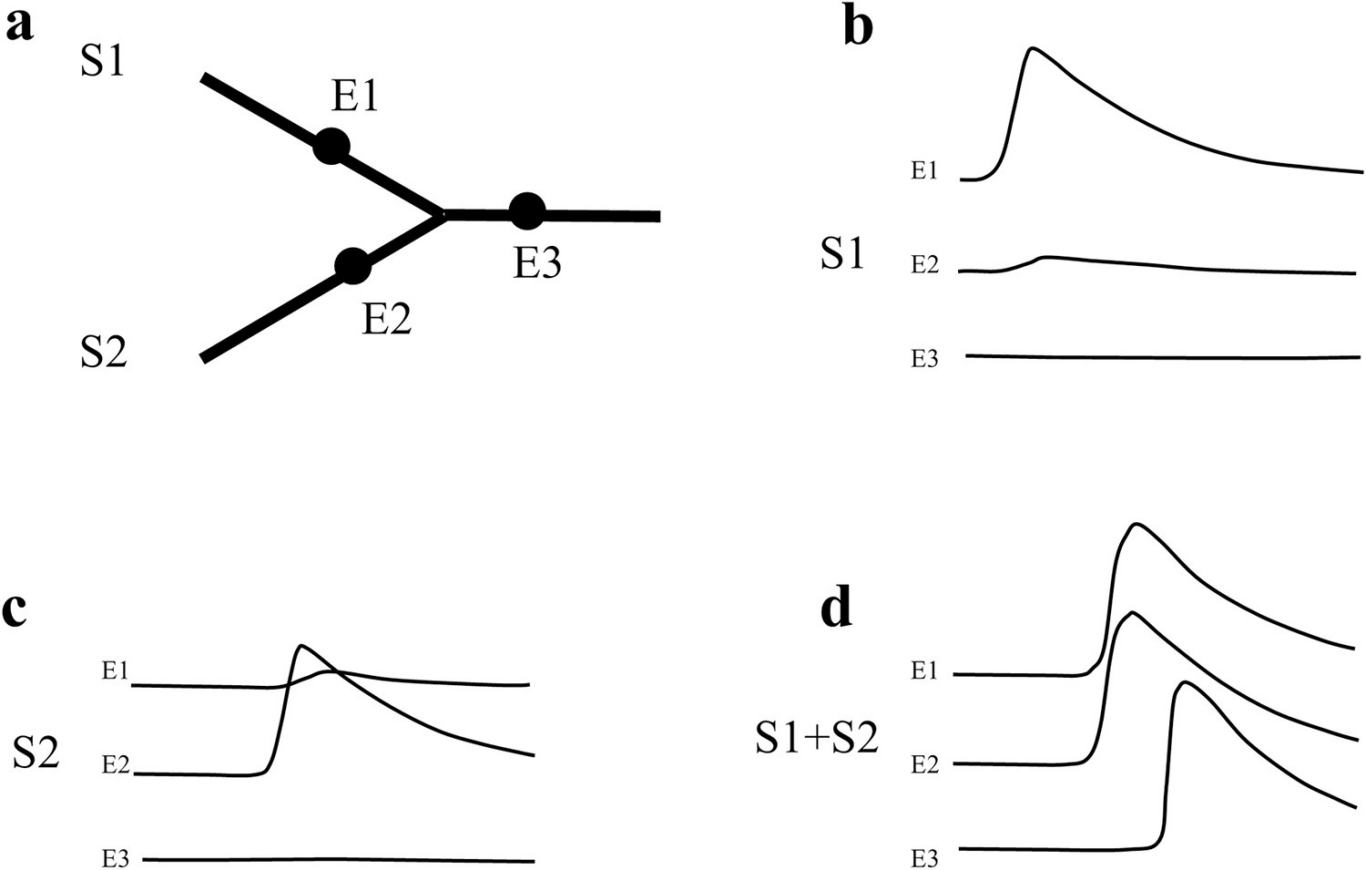
Computer simulation of activation block and summation of cardiac activity in a converging geometry. Panel **a** shows three strands of excitable cardiac cells with stimulation sites S1 and S2, and recording sites E1, E2 and E3. In panel **b** only the upper strand is activated (S1). This results in conduction block at the intersection; there is no signal at E3 and only an electrotonic deflection at E2. In panel **c** the lower strand is activated (S2), which results in conduction block at the intersection as well. Only if both strands are activated (panel **d**, S1+S2), the exit path is excited (electrogram at E3). The figure is composed from data of van Capelle [47].

An example of summation of activation in cardiac tissue is illustrated in Fig 6. The figure shows two strands of excitable tissue that merge into a common exit path. If one strand is stimulated, activation arriving at the discontinuity has to deliver current to activate two other strands. If the strand is only able to deliver just enough current to activate one strand, activation will block at the intersection. However, if the two strands are activated simultaneously, enough current can be delivered due to summation, and the exit path (the third strand) will thus be activated. The figure is derived from data of van Capelle based on a computer model of coupled excitable elements [47, 48].

Summation of activation in heart tissue has been reported in several studies. Evidence suggesting that summation could be a feature of atrioventricular (AV) nodal conduction has been reported by Hoffmann and Cranefield [49]. The same authors showed that summation of activation occurred in a common segment of a branched bundle of the false tendon, a geometry similar to the branching structures in *Mimosa* [50]. In their study, excitability at the common segment was, however, depressed by elevating potassium. Zipes et al. provided evidence for summation of activation in the rabbit AV node by creating two separated pathways to the node [27]. When premature stimuli were delivered at the two separated inputs of the AV node, at coupling intervals equal to the refractory period, conduction block occurred. Stimulating both inputs at the same time, however, resulted in propagation through the node.

The observations mentioned above indicate that structures consisting of separated pathways that merge into a common path may facilitate conduction by summation. The setup as used in our *Mimosa* experiments fits with this concept. Summation did, however, not always occur. This might be related to the fact that the time of activation cannot be regulated with great precision by the cold water drop procedure. Differences in arrival time at the summation site might also affect summation capabilities, as we showed in experiments with cardiac cell cultures.

The cardiac tissue culture observations indicate that structures consisting of separated pathways that merge into a common path may facilitate conduction by summation. The setup as used in our *Mimosa* experiments fits with this concept. Summation did, however, not always occur. This might be related to the fact that the time of activation cannot be regulated with great precision by the ice water drop procedure. Differences in arrival time at the summation site might well affect summation capabilities, as we showed in experiments with cardiac cell cultures. The measurements indicate that minimal delay between the arrival time of activations at discontinuities is critical for obtaining summation of activation and this may well explain the failures of activation in this study of *Mimosa*.

Although our data indicate that summation of activation can occur in *Mimosa*, the way summation arises at the petiole-branch interface may differ from the cell culture experiments, because it is not entirely clear how different pathways are used in *Mimosa*. As shown by Lautner APs and VPs may, at least partly, use the same pathway [35]. Data suggest, however, that summation of AP and VP activation results in enough activating current to pass the petiole-stem discontinuity. This is supported by the observation that time delay between AP and VP plays a crucial role. A large delay after mechanically cutting leaves, resulted in failure of activation beyond the interface. This is similar to the observation that conduction block occurs in the cardiac cell cultures if the delay in activation of adjacent tracts exceeded a certain value.

### Comparative considerations

*Dionaea muscipula* uses -temporal- summation of activation to capture insects, to obtain nutrients that are not available in the soil of the swampy areas in which they live [51]. Their leaves contain glands that secrete enzymes to digest the captured insects. Leaves can collapse quickly and function as a trap for their prey. The leaf epidermis has touch- sensitive hairs that detect movement of an insect. If a single hair is displaced, fast electrical depolarization is initiated, but leaves do not collapse. However, a second touch of the hair within 20 seconds will close the trap, which avoids ineffective closure of the trap and saves energy. Because delay between activation can be as large as 20 seconds, this kind of summation seems to be unlikely as an explanation for summation in the *Mimosa* plant.

*Mimosa pudica* uses different types of electrical activity to respond to environmental stimuli. For non-damaging stimuli, a local response is usually sufficient, whereas a damaging stimulus will require a more global reaction for optimal protection of the plant. A global reaction requires more energy, so the difference in response is likely related to a cost-benefit balance. *Mimosa* controls the difference in reaction to a stimulus by APs or VPs that respectively block or favor activation propagation at the petiole-stem interface. Our data suggest that VP activation alone is often not sufficient for passage of activation at the interface, but that summation of AP and VP activity is needed.

Summation of activation is a phenomenon that can arise in healthy myocardial tissue (AV node), but it may also play a role in arrhythmia generation in cardiac disease. Thus, summation can be beneficial as well as harmful in the heart. Its function is less clear for *Mimosa*. Electrical activity in higher plants has been shown to be transmitted via phloem vessels, but parenchymal cells have been documented to participate in conduction of VP activity as well. Therefore, it is not clear whether separate pathways are used or whether the two activations summate within the same pathway. Data also show that delay between the two activations plays an important role in both cardiac tissue and in *Mimosa*.

In this study, extracellular electrograms were used. The electrograms recorded in *Mimosa* differ from electrograms obtained from mammalian myocardium. Extracellular electrograms recorded in heart tissue at sites where activation passes are mainly biphasic, whereas electrograms recorded from *Mimosa* are virtually negative only. This difference is mainly based on the difference in upstroke velocity of the APs that are much faster in heart cells than in plant cells. Although depolarization in heart cells is mediated by sodium current and by calcium and chloride current in plant cells, basic electrophysiological characteristics are similar. The electrical circuit for coupled cardiac cells as described by Kucera et al. [52] is also similar to the electrical circuit for the pulvinus of *Mimosa* as proposed by Volkov et al. [36]. Therefore, the mechanism of summation via loading of the membrane capacitance by the two types of activation in *Mimosa* seems to be similar to the cardiac mechanism. In addition, activation of the (compact) AV node of mammalian hearts is mainly based on calcium ions and AV nodal APs have low upstroke velocities.

To assess whether ageing affected electrical activity we assessed conduction velocity during the first and last months of the recordings. Recordings were carried out during a period of 3 months, with first recordings starting 3 months after seeding. For recordings made during the first month mean conduction velocity was 2.0 and 2.1 cm/s for drop- and flame-induced activity respectively. These values reduced to 1.0 and 0.8 cm/s during the last month. The difference in conduction velocity between the young (early phase) and old (late phase) petiole is statistically significant. Because the plant was its own control, age did not influence the outcome.

## Conclusions

We measured unipolar extracellular electrograms from petioles and stems of *Mimosa* plants after cold- or heat-induced activation. Our experiments showed that VPs were preceded by APs and that the occurrence of both types of activation were required to allow electrical activation to pass the petiole-stem interface. By stimulating petiole and stem simultaneously we demonstrated that summation of activation is indeed possible in *Mimosa*. Using branching cultures of heart cells, we showed that summation of activation occurs at tissue discontinuities and that two stimuli at different branches can elicit a generalized response also if a small time delay between the stimuli is present. The observations indicate that summation occurs in branching cardiac structures and suggest that summation of activation plays a role in the propagation of nocuous stimuli in *Mimosa* branches.

## Materials and methods

### Plants

*Mimosa pudica* plants were grown from seeds (EAN code: 8711117555001) obtained from Horti Tops, Tuinplus, Heerenveen, The Netherlands. Plants were grown at room temperature in containers filled with potting soil. After four weeks in the grow room, seedlings of each plant were transplanted. Experiments were performed in the experimental laboratory on > 3 month old plants. All experiments were performed in accordance with relevant guidelines and regulations.

### Electrical recordings

Unipolar extracellular electrograms were recorded from the stem, petioles and pulvini of 5 specimens. Recordings were made with respect to a reference Ag/AgCl electrode inserted in the soil at about 2 cm from the stem. Recording electrodes consisted of isolated silver wires (diameter 100 μm) of which 5 mm of the insulation from the tip was removed. The bare end of the wire was hooked and hung around the petiole, pulvinus or stem and connection with the tissue was made with conductive gel (Ultra/Phonic, Codali, Brussels). The advantage of this concept is that no wound has to be made for impalement and that the electrode is able to follow movements of the petiole and pulvinus. The recording electrode was connected to the positive input of an instrumentation amplifier, whereas the reference electrode was connected to the negative input. Three electrograms were recorded simultaneously with a National Instruments USB-6009 data acquisition system (at 1 kHz sampling rate). In 7 experiments, the 3 electrodes were arranged in a row on the petiole. In 8 other experiments, 2 electrodes were on the petiole and 1 on the stem.

### Induction of electrical activity

Petioles and/or stems were electrically activated by a drop of melting ice water (4°C). A single melting water drop was applied to a petiole and/or stem using a disposable 5 ml pipette. With this pipette water drops of approximately 100 μl were produced in a reproducible manner. The water drop attached to the petiole or stem and its local cooling effect induced electrical activation. In case both a petiole and the corresponding stem had to be activated simultaneously, two pipettes were used. There was virtually no delay between the application of the droplets at the two locations. Because conduction velocity could have been different in petiole and stem, a time delay may have occurred between the arrival of the two activation fronts at the bifurcation. We therefore measured the difference in arrival time of activation at the petiole and stem electrode and determined whether conduction or conduction block occurred at the interface.

Heating of pinnules or petioles was performed with a lighter flame for 1 second, whereas 2–4 mm long incisions in leaves were made by a small scissor.

### Experiments

To determine the difference in extracellular electrograms between cold water and flame- induced activation, the same site of a petiole was activated twice. First, recordings were made from 3 sites after applying the water drop. Then, a period of 30 minutes was introduced for recovery, after which a flame was applied to activate the petiole again, and a second recording was made (n = 15). Summation of activation was tested by activating the petiole and stem simultaneously with drops of cold water (n = 19). Mechanical cutting of leaves was performed to obtain electrograms with larger delays between AP and VP components (n = 5).

### Cultured heart tissue

Neonatal rat heart cells were isolated and grown in star-shaped patterns with 8 arms, 0.1 mm wide and 1.6 mm long (Fig 5), as described before [53]. These cells grow and couple electrically by making connexins (protein channels), comparable to plasmodesmata in plant cells. Extracellular unipolar electrograms were recorded at several sites in the tracts. Pacing was performed by electrical stimulation at twice diastolic current threshold with a continuous drive train. The cycle length during stimulation at the tracts was reduced till 2:1 activation block at the tract-star center interface occurred. This partial conduction block is because at high stimulation frequencies recovery from inactivation of the sodium channels becomes impaired [54]. This reduces current availability and leads to activation block at the star entrance. Because the next beat has more time for recovery, more current is generated and activation may pass the tissue discontinuity again, resulting in 2:1 block.

### Neonatal rat cardiomyocytes

Animal experiments were approved by the local Animal Experiments Committee (Academic Medical Center, University of Amsterdam) and carried out in accordance with governmental and institutional guidelines and regulations. The study was conducted in accordance with the Declaration of Helsinki as revised in 2013. Methods are reported in accordance with ARRIVE guidelines.

To isolate neonatal rat heart cells, hearts were rapidly explanted from 1 to 2-day old Wistar rats. Care was taken to minimize the stress of the animals. Neonatal rats were covered with insulation material during transportation, to prevent cooling. Pups were decapitated using sterile scissors. No anesthesia was used, because pain related action potentials in rat pups only occur after day 12 of birth [55]. Discomfort for the animals is very short and heads were immediately submerged in liquid nitrogen. The chest was opened along the sternum to allow access to the chest cavity and the heart. Hearts were extracted from the body with sterile scissors and the ventricles were cut into pieces and dissociated with trypsin (1 mg/mL; Becton Dickinson BV, Breda, The Netherlands) and collagenase type 2 (1 mg/mL, Worthington Vollenhove, The Netherlands, 230 units/mg). A pre-plating step was performed to minimize fibroblast contamination. Neonatal rat heart cells were plated and grown in star-shaped patterns with 8 arms provided with extracellular electrodes.

### Statistics

Statistical analyses of extracellular recordings of *Mimosa* were performed by paired t-tests, to compare electrogram characteristics after cold and heat stimulation. Mean values and standard deviations of various variables are displayed in Table 1. Differences were regarded statistically significant at p≤0.05.

## Supporting information

S1 Table (XLSX)
